# Novel Combination of Impella and Extra Corporeal Membrane Oxygenation as a Bridge to Full Recovery in Fulminant Myocarditis

**DOI:** 10.1155/2012/459296

**Published:** 2012-07-10

**Authors:** Sachin Narain, Gian Paparcuri, Thomas M. Fuhrman, Richard B. Silverman, William T. Peruzzi

**Affiliations:** Department of Anesthesiology, Perioperative Medicine and Pain Management, University of Miami Miller School of Medicine, 1611 NW 12th Avenue (C-301), Miami, FL 33136, USA

## Abstract

A 31-year-old male was transferred to our hospital with severe heart failure due to viral myocarditis. He progressed to multiorgan failure requiring intubation and maximal doses of multiple vasopressors. Circulatory support was provided with an Impella device as a bridge to an extracorporeal membrane oxygenation (ECMO) system. On full mechanical cardiovascular support, the patient's hemodynamic status improved and ECMO and Impella were explanted after 48 hours. Three days later, he was extubated and continued on to a full recovery. There are no specific therapies for fulminant myocarditis but first-line treatment is supportive care. ECMO is commonly used in patients with severe heart failure. In severe systolic dysfunction, left ventricular decompression is required to reduce myocardial wall stress, decrease myocardial oxygen requirements, and enhance the chances of recovery. The Impella, an active support system, is less invasive than classical decompressive techniques and is associated with lower requirements for blood products with fewer thromboembolic complications. This is the only case reported of the contemporary use of Impella and ECMO as a bridge to full recovery in an adult with myocarditis. It also presents a novel use of the Impella device in decompressing the left ventricle of an adult patient on ECMO.

## 1. Introduction

Management of cardiogenic shock secondary to fulminant myocarditis can be quite complex. Supportive care is a mainstay of treatment; and in certain cases, this may include technologies such as extracorporeal membrane oxygenation (ECMO), intraaortic balloon pump or Impella implantation. In this case, we describe the management of an adult patient with fulminant myocarditis utilizing the novel combination of ECMO and Impella support.

## 2. Case Presentation

A 31-year-old man with no prior medical history presented to his primary care physician with three days of high fever, sore throat, cough and diarrhea. Initial exam revealed tachycardia, bilateral conjunctival injection, an erythematous oropharynx, cervical adenopathies and right lower quadrant abdominal tenderness. 

His symptoms persisted despite antibiotic therapy. Upon presentation to an outside emergency room, laboratory workup revealed an elevated white blood cell count with left shift, isolated troponitis, and acute renal and hepatic failure. He had bibasilar pulmonary infiltrates and hepatomegaly on imaging.

The patient became hemodynamically unstable—requiring pharmacological support with multiple agents. He was initially transferred to our coronary care unit with a diagnosis of cardiogenic and distributive shock secondary to viral myocarditis. Transthoracic echocardiography (TTE) reported an estimated ejection fraction (eEF) of 10% with biventricular distention and severe mitral regurgitation. Endocardial biopsy revealed a florid round cell inflammation consistent with viral myocarditis. The patient was treated with steroids, broad-spectrum antibiotics, antivirals, and intravenous immunoglobulin. Cardiogenic shock progressed rapidly to multiorgan failure. The patient became anuric and sustained shock liver with a severe coagulopathy and also required tracheal intubation and mechanical ventilation due to worsening pulmonary edema.

 Given his deteriorating condition and escalating doses of three different inotropes and vasopressors, further circulatory assistance was provided with an Impella Recover LP 2.5 device (Abiomed, Danvers, Mass). It was implanted via the left femoral artery under fluoroscopic guidance and provided a flow rate of 2.5 L/min. For the first 12 hours postimplantation, his hemodynamic indexes all showed a marked improvement and vasopressor infusions were titrated down (Table 1).

Unfortunately, despite the temporary improvement, his condition began to deteriorate after 12–14 hours. He again developed a worsening lactic acidosis and hypoxia; and he required maximal doses of four different vasoactive drugs to maintain his blood pressure and cardiac output. His poor prognosis was discussed with his family along with the possibility of utilizing extra corporeal membrane oxygenation (ECMO) as a temporary measure to allow his organs to recover. Ultimately, the patient was transferred to the surgical intensive care unit for emergent bedside peripheral venoarterial (VA) ECMO implantation via the right femoral vessels. With full mechanical support, the patient's hemodynamic indexes immediately improved and vasoactive requirements decreased significantly. ECMO flow was set to 4 L/min and the Impella output was decreased to 1 L/min to maintain left ventricle unloading. His systolic blood pressure increased from 60 mmHg to 90–100 mmHg and the doses of the pressors were all immediately weaned down. Continuous renal replacement therapy circuitry was attached to the ECMO circuit as he remained oliguric.

In the following 48 hours, the patient's condition improved dramatically. Oxygen delivery increased, metabolic acidosis subsided, and pharmacological cardiovascular support was tapered off. Bedside TTE showed a hyperdynamic, vigorously contracting left ventricle with an eEF above 50%. He also had mild aortic insufficiency which was likely due to the presence of the Impella device. The patient's hepatic function also improved, demonstrated by increased anticoagulant requirements for ECMO.

At this point, ECMO and Impella were both explanted successfully at the bedside under transesophageal echocardiographic guidance. A hemodialysis catheter was placed in the left groin and he was transitioned to renal replacement therapy with continuous venovenous hemodialysis which was tapered off as his urine output improved. 

He completed a two-week course of antimicrobials and his blood cultures remained negative for any other pathogens. He was extubated a few days after mechanical cardiovascular support was discontinued. The patient was transferred to the inpatient rehabilitation center for further care of his ICU acquired neuropathy. 

## 3. Discussion

Myocarditis accounts for 10% of patients with acute onset heart failure and up to 20% of sudden death among young adults. Patients with the severe form, or fulminant myocarditis, often present in cardiogenic shock. Chances of recovery increase with early recognition and aggressive treatment. There are no specific therapies for fulminant myocarditis described in the literature. The first line treatment is supportive care, inotropic support, and, if necessary, mechanical circulatory support [1].This patient had a pronounced multiorgan failure secondary to cardiogenic shock. The early institution of aggressive support coupled with his excellent functional status prior to this illness led to a rapid and full recovery.

The Impella LP 2.5 left ventricle assist system is an axial flow pump that is positioned across the aortic valve and provides up to 2.5 L/min of flow removed from the LV which is then pumped into the ascending aorta (aortic root). It is able to provide support without the need for cardiac synchronization, which can be erratic during patient transport or in the presence of cardiac arrhythmias.

Compared with other devices used individually to provide circulatory support, like ECMO, the Impella is a less invasive option associated with lower requirements for blood products and fewer thromboembolic complications [2]. And while an intraaortic balloon pump is an effective assist device with a good safety record, it is a passive support system and is unable to affect preload [3]. 

Impella use is steadily becoming more widespread. Its use has been described in the management of postcardiomiotomy heart failure and during high-risk percutaneous coronary interventions [4]. There is one reported pediatric case where an Impella 5.0 was used as a bridge to full recovery from fulminant viral myocarditis [5]. 

In patients with severe cardiopulmonary failure, VA ECMO is an option. However, in the face of severe systolic dysfunction, left ventricular decompression (aka unloading or venting) is often required to reduce myocardial wall stress, decrease myocardial oxygen requirements, and enhance the chances of ventricular recovery [6]. 

Unloading during ECMO support historically has been achieved surgically with a cannula placed in the left atrium, or percutaneously with an atrial septostomy. In our case, the Impella was a valuable alternative to more classical, and invasive, decompressive techniques. The Impella can be inserted percutaneously at the bedside under echocardiographic or fluoroscopic guidance, avoiding the need for a thoracotomy. A similar use of the Impella has been described in pediatric cases of congenital heart disease [6, 7]. 

For this patient, the addition of ECMO resulted in a 4 L/min flow of well-oxygenated blood in a retrograde fashion from the right femoral vessels up the thoracic aorta to the aortic arch. A distal side port was attached to the arterial cannula so oxygenated blood could be supplied to the right lower extremity itself. Poorly oxygenated blood passing through the pulmonary circulation to the LV was then pushed forward by the Impella's 1 L/min flow rate along with the patient's own intrinsic cardiac output. 

To verify that he had adequate cerebral oxygen delivery, arterial blood gas measurements were drawn from the right upper extremity as its blood supply would be the most proximal to the heart and, therefore, the closest to the source of poorly oxygenated blood. Blood gases drawn from the right upper extremity were adequate, concluding that cerebral oxygen delivery was sufficient. Ultimately, he did not have any major neurological complications. 

In our patient, the Impella device was initially implanted for circulatory compromise. Once the patient was started on ECMO, the preexisting Impella was used to decompress the left ventricle. In the future, when starting a critically ill patient on ECMO, it may indeed be beneficial to consider concurrent Impella implantation.

In this case report, we describe a new potential function of the Impella in the adult population. To our knowledge, this is the first case reported of contemporary use of the Impella and ECMO in an adult as a bridge to full recovery for fulminant myocarditis. This case also presents a novel use of the Impella device in decompressing the left ventricle for an adult on EMCO support.

## Figures and Tables

**Table 1:** Changes in hemodynamic parameters following Impella implantation.

Index	Pre-Impella	Post-Impella
MAP (mmHg)	47	66
CVP (mmHg)	14	22
CI (L/min/m^2^)	2.9	4.0
SVR (dyn-s-cm^−5^)	600	300

All hemodynamic parameters improved in the initial post-implantation period. For the pre-Impella values, the patient was still on multiple vasopressor agents. For the post-Impella values, vasopressors had been nearly titrated off. (MAP: mean arterial pressure, CVP: central venous pressure, CI: cardiac index, SVR: systemic vascular resistance).
